# The Troubles of the Masseuses

**Published:** 1899-05-27

**Authors:** 


### The Hospital

A Journal of

The Medical Sciences and Hospital Administration.

Vol. XXVI.—No. 661.] Edited by Sir Henry Burdett, K.C.B., and Solomon C. Smith, M.D., M.R.C.P. [May 27, 1899.

### The Troubles of the Masseuses.

The development of the art of massage and its growing recognition by the profession as an important method of treatment in a considerable range of diseases have led a number of most respectable people to take it up as a means of earning their living, and it has fallen upon these people as a heavy blow and great discouragement that, in consequence of the evil deeds of certain persons, who might well have received the kind attentions of the police long before they did, the word massage has come to connote in the eyes of the public much that is abominable, and the title of masseuse to suggest quite the opposite of what is proper and respectable. It is a great hardship, and the question is—What is to be done? One thing is certain, massage must not be allowed to go. In the daily work of the physician and the surgeon massage has come to occupy a position of importance which could hardly have been predicted for it a few years ago, and in the management of many cases the work of the masseur and the masseuse has become an essential auxiliary to medical treatment. Under these circumstances we can hardly feel surprised that those who have devoted time and money to their training, and are conscious not only of the honesty of their work but of its great utility, should feel aggrieved at the present condition of affairs, and should demand that their title should be protected, that they should become a close corporation, admission to which should only be obtained after proper training, and that a register should be established to which the public could refer, and entry upon which should be restricted to those who had given evidence of training, of skill, and of respectability. We do not feel surprised at the demand, yet we can give no sanction to any such scheme.

In a sense the difficulty is the same as that of the midwives and the spectacle makers over again, but there is this difference, that its solution by "registration" is a much more serious business, that every objection that has been raised against the legal registration of midwives can be raised with infinitely greater force against the registration of masseurs, and that there is not the same plea of giving protection to the poor to be raised in its favour. The midwife and the spectacle maker have their field of work strictly limited and cut out for them, but the field of the masseur is the whole of the human body, and there are not lacking people who are ready to maintain that it covers almost every disease to which the human body is liable. Those who ask for the legal registration of "qualified" masseurs have then to face this, that they are asking for the legal recognition of people as being qualified to treat disease by their own special methods, although they have received no complete medical education. The only reasonable way out of the difficulty seems to lie in the maintenance of the broad principle which has received the sanction of the General Medical Council, namely, that before any person undertakes to treat disease in any part of the body he ought to give proof of having studied the structure and functions of the body as a whole, both in health and in disease. It is open to anyone after qualification to take up any specialty that he pleases, but a minimum of knowledge should be required of him, and that minimum cannot be allowed to be placed at a lower level than that which is demanded from qualified medical practitioners. What, then, about the midwives, masseurs, and spectacle makers, all of whom are anxious to be regarded as "qualified" in their own particular departments? We can only repeat that all treatment of disease falls properly to those who have taken the pains to render themselves legally qualified for that work, and that although certain modes of treatment, such, for example, as massage, can best be carried out by people who devote themselves especially to one or another of these methods, these people should all work under the direct control of qualified medical practitioners, who should be responsible for all that is done. No one could consider it a satisfactory arrangement which would allow a physician to prescribe massage, and leave it open to his unfortunate patient to have to wander about Piccadilly to find someone to carry out the treatment! As a matter of fact this is not done. All the best massage work is done under the direct control of the physician who prescribes it, and this ought to be the rule throughout. Under such circumstances a public "register" is quite unnecessary. The midwives have a strong claim to registration so long as they keep to their proper work of attending natural confinements, first on account of this being a natural process, and, second, because the sympathy of all good men goes out to women in their hour of peril, and it is difficult to refuse anything which might add to their safety. But these other specialties stand quite on a different footing; they are all departments of medical practice, and should be under the responsible control of medical practitioners.

